# Patient Health Literacy and Communication with Providers among Women living with HIV: A Mixed Methods Study

**DOI:** 10.1007/s10461-021-03496-2

**Published:** 2021-10-12

**Authors:** Henna Budhwani, C. Ann Gakumo, Ibrahim Yigit, Whitney S. Rice, Faith E. Fletcher, Samantha Whitfield, Shericia Ross, Deborah J. Konkle-Parker, Mardge H. Cohen, Gina M. Wingood, Lisa R. Metsch, Adaora A. Adimora, Tonya N. Taylor, Tracey E. Wilson, Sheri D. Weiser, Oluwakemi Sosanya, Lakshmi Goparaju, Stephen Gange, Mirjam-Colette Kempf, Bulent Turan, Janet M. Turan

**Affiliations:** 1. University of Alabama at Birmingham (UAB), Birmingham, AL 35294; 2. University of Massachusetts Boston (UMB), Boston, MA 02125; 3. TED University, Ankara, Turkey; 4. Emory University, Atlanta, GA 30322; 5. Baylor College of Medicine, Houston, Texas 77030; 6. University of Mississippi Medical Center (UMMC), Jackson, MS 39216; 7. Stroger Hospital of Cook County, Chicago, IL 60612; 8. Columbia University, New York, NY 10027; 9. University of North Carolina at Chapel Hill, Chapel Hill, NC 27599; 10. State University of New York, Downstate Health Sciences University, Brooklyn, NY 11203; 11. University of California, San Francisco, San Francisco, CA 94143; 12. Montefiore Medical Center, New York, NY 10467; 13. Georgetown University, Washington DC 20007; 14. Johns Hopkins University, Baltimore, MD 21287; 15. Koc University, Istanbul, Turkey

**Keywords:** Health Communication, Health Literacy, HIV, African American, Latina

## Abstract

In this mixed-methods study, we examine the relationship between provider communication and patient health literacy on HIV continuum of care outcomes among women living with HIV in the United States. We thematically coded qualitative data from focus groups and interviews (N=92) and conducted mediation analyses with quantitative survey data (N=1,455) collected from Women’s Interagency HIV Study participants. Four qualitative themes related to provider communication emerged: importance of respect and non-verbal cues; providers’ expressions of condescension and judgement; patient health literacy; and unclear, insufficient provider communication resulting in diminished trust. Quantitative mediation analyses suggest that higher health literacy is associated with higher perceived patient-provider interaction quality, which in turn is associated with higher levels of trust in HIV providers, improved antiretroviral medication adherence, and reduced missed clinical visits. Findings indicate that enhancing provider communication and bolstering patient health literacy could have a positive impact on the HIV continuum of care.

## RESUMEN

En este estudio de métodos mixtos, examinamos la relación entre la comunicación del proveedor y la alfabetización sanitaria del paciente sobre los resultados de la atención continua del VIH entre las mujeres que viven con el VIH en los Estados Unidos. Codificamos temáticamente datos cualitativos de grupos focales y entrevistas (N=92) y realizamos análisis de mediación con datos de encuestas cuantitativas (N=1,455) recopilados de participantes del Estudio de VIH entre agencias de mujeres. Surgieron cuatro temas cualitativos relacionados con la comunicación con el proveedor: la importancia del respeto y las señales no verbales; las expresiones de condescendencia y juicio de los proveedores; alfabetización en salud del paciente; y una comunicación poco clara e insuficiente con el proveedor que da como resultado una disminución de la confianza. Los análisis de mediación cuantitativa sugieren que una mayor alfabetización en salud se asocia con una mayor calidad de interacción percibida entre el paciente y el proveedor, que a su vez se asocia con niveles más altos de confianza en los proveedores de VIH, una mejor adherencia a la medicación antirretroviral y una reducción de las visitas clínicas perdidas. Los resultados indican que mejorar la comunicación con los proveedores y reforzar la alfabetización sanitaria del paciente podría tener un impacto positivo en la atención continua del VIH.

## INTRODUCTION

Clear and non-judgmental provider communication is a key component of quality healthcare.^1–3^ Studies have shown that when aspects of provider communication such as unclear medical guidance or use of clinical terminology without explanation, the likelihood that patients will be non-adherent increases.^1–3^ Relatedly, when patients feel stigmatized by a provider’s verbal or non-verbal communication, they are less likely to follow their recommendations and to trust these providers, and are more likely to miss clinical visits, particularly when seeking healthcare for a stigmatized condition such as human immunodeficiency virus (HIV) infection.^4–7^

Women represent nearly a quarter of the people living with HIV in the United States (US).^8^ African American and Latina women living with HIV (WLHIV) are less likely to use antiretroviral medications, less likely to be virally suppressed, and more likely to die due to HIV-related complications, as compared to their White counterparts.^9–11^ Structural forces, such as stigma, racism, and misogyny -- which influence access to educational and economic opportunities, while reducing community standing and social capital -- harm women of color.^12–14^

Poor provider communication may exacerbate feelings of low self-worth among populations that routinely experience intersectional stigma (stigma related to holding multiple characteristics that are devalued by society), such as WLHIV who may experience stigma related to being a woman (misogyny and sexism), being a person of color (racism), socioeconomic status (classism) and living with HIV (having a stigmatized medical condition).^15–17^ While clinical settings should be pro-patient and non-judgmental; this is not always the case. In the United States, women of color, who are disproportionately represented within the community of WLHIV, routinely report receiving poor healthcare and having worse health outcomes than their racial minority counterparts.^12,18,19^

Considering that negative stereotypes associated with WLHIV and people of color may have been long held by providers, and that the healthcare financing system rewards quick visits and efficient clinical delivery,^20^ enhancing provider communication continues to be an unmet clinical and public health goal. In 2003, the Institute of Medicine issued a report stressing the importance of appropriate communication between providers and patients, as a mechanism to improve the healthcare quality for patients of color.^21^ Yet, eighteen years later, gaps in communication persist, and patients of color often experience stressful encounters in healthcare settings related to poor, insensitive, abrupt, or insufficient communication.^22–24^

Low patient health literacy is a barrier to effective patient-provider communication. Health literacy is the extent to which a patient has the capacity to understand (and obtain) relevant health information.^21,25,26^ Within this construct is the patient’s ability to understand her diagnosis (e.g., HIV infection), make appropriate decisions about her health (e.g., adhering to antiretroviral medications), and includes her comprehension of her disease and necessary treatment.^27^

Considering the potential interplay between provider communication and patient health literacy on the delivery of quality healthcare and achievement of optimal health outcomes, we conducted a mixed-methods study to elucidate this relationship among WLHIV. In this study, we utilized data collected from women enrolled in the Women’s Interagency HIV Study (WIHS),^28^ a national cohort study in the United States designed to better understand the impact of HIV disease on women.

## METHODS

### WIHS network and study design

Established in 1993, the WIHS is a multi-site, prospective longitudinal cohort study that captures the experiences of WLHIV and women who are at risk for HIV acquisition to investigate the clinical progression of HIV infection as well as conduct epidemiological and behavioral studies of high public health priority.^28^ Data collection for the WIHS occurrs via interviews, standardized surveys, physical assessments, and laboratory tests. This sub-study leverages a convergent parallel mixed methods design;^29^ and data for this sub-study were collected between November 2017 and December 2018.

### Qualitative data

#### Study design

We conducted twelve focus groups with minority (African American and Latina) WLHIV, each with 5-11 participants, supplemented with a small set of 3 interviews for exclusively Spanish-speaking participants (N=92) across six WIHS sites in Georgia, Alabama, New York, North Carolina, Illinois, and Mississippi. Using the focus group format as our primary mechanism for data collection enabled us to collect data that benefitted from intergroup discussion regarding topics of interest and allowed study participants to expand upon one another’s comments and shared experiences, revealing areas of commonality as well as personal experiences where views diverged.

#### Recruitment and study participants

All study participants were 25 years of age or older. Written informed consent was obtained from all focus group and interview participants. Participants received a $50 incentive. The study was approved by Institutional Review Boards at all participating sites.

#### Data collection

Once participants were consented, trained female focus group moderators conducted all sessions. Focus group guides were informed by prior WIHS findings and academic literature on the experiences of WLHIV; topics covered in these guides included patient-provider communication, experiences of stigma, and resilience. Focus groups were conducted in English; interviews were conducted in Spanish. Focus groups and interviews were conducted at research sites where WIHS study visits occurred. Both focus group discussions and interviews utilized the same semi-structured guide, enabling us to merge findings. At the beginning of all sessions, the interviewer or moderator provided this definition of healthcare provider: “By a healthcare provider, I mean a doctor, nurse practitioner, nurse, physician’s assistant, social worker, pharmacist, dentist, or other person that provides you with services at a doctor’s office, hospital, clinic, or pharmacy.” The guides were designed to elucidate experiences and opinions related to provider communication, patient health literacy, and patient engagement in HIV care. Women were encouraged to talk about their different healthcare providers during the sessions, including both HIV care and non-HIV providers, with some questions specifying their “main HIV care provider”. Findings regarding other aspects of quality of care discussed by the participants are presented elsewhere.^8^

#### Data analysis

Focus groups and interviews were audio recorded; an independent transcription company professionally transcribed these recordings verbatim. Spanish language interview transcripts were translated into English for analysis. Transcripts were analyzed using a two-stage inductive thematic analysis process. In stage 1, a team of five researchers collaboratively developed a codebook that included broad and fine codes to classify data that aligned with patterns that emerged during initial review, reflected the data collection objectives from the study’s aims, and considered prior relevant research. Once this baseline set of codes were finalized, a sub-set of the study team coded the transcripts in the NVivo 12 qualitative analysis software. A set of transcripts were first double coded, then coded transcripts were compared for congruence and inconsistencies, and finally, the full team reconciled and resolved discrepancies through facilitated discussion to improve reliability. In stage 2, the team refined the codebook and applied it to all of the transcripts. The researchers reviewed coded transcripts to identify emergent themes from the data, and their findings were presented to senior investigators for feedback prior to finalization.

### Quantitative data

#### Study design and data collection

We employed a cross-sectional design for the quantitative portion of this study; study participants were WLHIV who were enrolled across the nine WIHS sites located in New York, Washington DC, Illinois, California, Georgia, Alabama, Mississippi, Florida, and North Carolina (N=1,455). All participants were adults aged 18 years and older and were English speaking. The Institutional Review Board at each site approved study procedures. All participants provided written informed consent.

#### Measures

Validated self-administered measures assessed health literacy, patient-provider interaction quality, trust in HIV care providers, HIV visit adherence, and antiretroviral medication adherence.

##### Health Literacy.

Health literacy was assessed with three questions from the Brief Health Literacy Screen^30^ (i.e., “How confident are you filling out medical forms by yourself?”, “How often do you have someone help you read hospital materials?”, and “How often do you have problems learning about your medical condition because of difficulty understanding written information?”) rated on a five-point scale (extremely to not at all for the first item and all of the time to none of the time for the other two items), with higher scores indicating higher health literacy (The first item was reversed). In the present study, Cronbach’s alpha was .75.

##### Patient-Provider Interaction Quality.

This was assessed using the 16-item Interpersonal Processes of Care Survey.^31^ Items are rated on a five-point scale ranging from 1 (never) to 5 (almost always), with higher scores indicating more positive (better) interaction with HIV care providers (some items were reverse coded to maintain this directionality). Sample items include the following: “How often did HIV care providers speak too fast?”, “How often did HIV care providers take your health concerns very seriously?”, and “How often did you and your HIV care providers work out a treatment plan together?” In the current study, Cronbach’s alpha was .90.

##### Trust in HIV Care Providers.

We assessed trust in one’s HIV care provider using the 8-item Safran Physician Trust Subscale of the PCAS.^32^ Items (e.g., “Your HIV care providers would always tell you the truth about your health, even if there was bad news” and “If a mistake was made in your treatment, your HIV care providers would try to hide it from you”) are rated on a five-point scale (strongly disagree to strongly agree), with higher values indicating higher trust in HIV care providers. In this study, Cronbach’s alpha reliability coefficient was .82.

##### Antiretroviral (ART) Adherence.

ART adherence was assessed with the single question asking participants to report “How often they took antiretrovirals as prescribed over past 6 months” which includes the following response options: “100% of the time”, “95-99% of the time”, “75-94% of the time”, “<75% of the time”, and “I didn’t take medications as prescribed.” As in previous studies using this measure, we employed the 95% cutoff as less than perfect adherence versus perfect adherence. Prior research has confirmed that this measure is a valid measure for treatment adherence.^33,34^

##### HIV primary care visit adherence.

This was assessed with a single question asking participants whether they “missed a regular HIV care appointment in the past 6 months.” We used it as a dichotomized variable, with 0 (missed at least one visit) versus 1 (attended to all scheduled visits). This measure has been shown to be a valid measure for HIV care engagement.^35^

#### Data analysis

In order to examine the associations between health literacy and patient-provider interaction quality, trust in HIV care providers, ART adherence, and HIV primary care adherence, three mediation analyses were conducted using the PROCESS macro for SPSS with 95% percentile confidence intervals (CIs) and 2,000 bootstrapping resamples. In these analyses, health literacy was the predictor, patient-provider interaction quality the mediator, and trust in HIV care provider, ART adherence, and visit adherence the outcomes. Covariates included the following: age, education, income, patient race, primary provider race, and illicit drug use (in past six months).

## RESULTS

### Qualitative results

#### Participant characteristics (N=92)

Participants identified as African American (89%) and Latina (11%). Over half (57%) were older than 50 years, reflecting the average age of the WIHS cohort. Sixty-five percent had been aware of their HIV diagnoses for over ten years; for 52%, the highest educational attainment was high school graduation. Half of participants (50%) were from Alabama, Mississippi, and Georgia. Over half (53%) had an annual income below the Federal Poverty Line for a single individual, which is $12,760 in 2021 ($12,140 in 2018).

#### Thematic findings

Key emergent themes related to patient-provider communication and health literacy were: 1) importance of respect and non-verbal cues; 2) providers’ expressions of condescension and judgement; 3) patient health literacy; 4) unclear, insufficient provider communication resulting in diminished trust.

#### Theme 1: Importance of respect and non-verbal cues

When WLHIV were asked about communicating with their providers, several identified cues that they perceived as discriminatory, disrespectful, or unsettling. Study participants consistently reported the importance of touch as a form of communication:

“Communication is the key. Don’t look down at us or question us as to why and this. No. Let’s be honest with one another. If you feel that you don’t wanna touch me or whatever, then that’s not your field.”

–New York

“This lady came in here. I asked her a question, and she said something. I said, ‘Excuse me?’ She said, ‘I’ll be right back,’ and when she came back in, my whole body was frozen, like there was nothing -- because when I came in, and I said, ‘Hello,’ to her and I gave her my hand, and she didn’t take my hand. I was frozen after that. I didn’t like her.

–New York

“Okay, when he walked in the room… we shook hands, which I always do… He wiped his hand on his pants, and then sat down. Whether it’s cuz I’m HIV positive, I’m black, you think I’m nasty, whatever it was. Every thought that went through my mind was negative. It was all inclusive. You are a Caucasian male. I’m an African American female. I’m HIV positive. You know my status. I don’t know yours. You wanna come in here and present yourself as being better than me? All of that went through my mind.”

–Alabama

In contrast, when providers used touch and other ways to connect with patients, they were perceived to be more capable, compassionate, and empathetic.

“One of my providers shook my hand, actually said, ‘Hi, [name]. How are you? How is your son [son’s name]?’ Those kinds of…those personal things, that really makes a big difference on your whole outlook when you’re going right to the doctor… you know what I’m saying?”

–New York

#### Theme 2: Providers’ expressions of condescension and judgement

While the focus group guide did not include specific questions about power dynamics, multiple examples and stories emerged that were illustrative of how providers exert their power and authority upon their patients through expressions of condescension and judgement.

“I had a provider to actually tell me if it weren’t for people like us, y’all wouldn’t get this care. I told her, ‘If it weren’t for people like us, y’all wouldn’t have a job.’”

–Georgia

“…Don’t tell me to turn around and be still and hold your head straight, ‘cause I’m finna stop you and I’m finna leave. You don’t talk to me like that. I’m your patient, you’re supposed to make me feel comfortable. I like to be comfortable, especially ‘cause I can’t see you in my mouth. I wanna be able to see what you’re doing to me. Don’t talk to me like I’m a child. I’m 40-years old, address me as such.”

–Mississippi

“I had unprotected sex again, caught something. I don’t know. [The provider] was just rude about it, about me catching something and not being careful. It was just the way she was talking to me, like she was downgrading me, so it just felt uncomfortable. After that, I wind up catching something again. Yeah, I thought I was being safe, but I wound up catching something again. It took me a while to actually go back to my doctor because I was scared I may get her, or they was gonna judge me…”

–Illinois

#### Theme 3: Patient health literacy

We found some evidence that patients’ health literacy may affect communication and understanding between patients and providers. This participant noted that she needed providers to “break down” the meaning of different laboratory tests for her.

“It’s all [providers] talk about, your viral load, your CD4 count, all that stuff. You need to break it down.”

–Alabama

In comparison, we found that our study participants took note of when their providers spent extra time to explain health conditions, new diagnoses, and medication considerations.

“Like I said, far as my healthcare provider, she is wonderful. Anything I wanna talk about and I can ask her about, she takes her time. She explains it. She break it down for me in layman term, so I can understand the way she’s talking to me. I love her.”

–Mississippi

“Now, my doctor… I love her, cuz she will break it down to the smallest term for me, and… she’s the one that taught me, when I go to the doctor’s office, what I should ask for. And I should remember the things that I need to ask for, you know… and just explain to me about the different medicine procedures, whatever I ask about. And stuff like that, so her and I, we communicate, we communicate real good.”

–Georgia

#### Theme 4: Unclear, insufficient provider communication resulting in diminished trust

On the other hand, our participants commented frequently on lapses in or insufficiency of provider communication, which diminished trust in their providers:

“Okay, I remember one time I came for labs. She didn’t tell me that I had to fast, so I came here for nothing, had to call the cab to go back home and make another -- reschedule another appointment. Then I told her, ‘Do I have to fast? Because you didn’t tell me that I have to fast. Do I have to fast?’ She goes, ‘Oh, yeah. You have to fast.’ Okay, thank you for sharing that with me.”

–New York

“The person that’s in charge forgot to tell me. I needed one more 40-minute appointment to be on the transplant list, but my doctors and all the surgeons thought I was on the list. It’s things like that when you can’t trust a facility…”

–North Carolina

While previous statements illustrate communication gaps that may have been due to an oversight or a provider assuming a level of health literacy, the quotation below indicates that this woman living with HIV felt her providers were deliberately hiding her medical information from her. This led her to mentally prepare for her clinical visits, armed with a series of questions to elicit information sharing.

“Some of the things are -- well, some of the doctors, it’s like if I’m not saying anything is wrong, then they just take it that nothing’s wrong. Sometimes, I wanna know -- I want the doctors to tell me. I have to question everything. Like, “Has my blood pressure been good? Does my liver look good? Does my kidneys look good?” I find myself trying to think of questions to ask them, that I feel like they’re not tellin’ me.”

–Alabama

### Quantitative results

#### Participant characteristics

Participants included 1,455 WLHIV aged between 28 and 83 years with a mean of 51.34 years (SD=9.13); 1,071 (73.6%) were African American, 211 (14.5%) White, and 146 (10.0%) other race/ethnicity, with the majority identifying their HIV providers as White (54.6%), reporting an average household income of $6,001-12,000 (35.3%), with nearly a third having completed high school (31.6%).

#### Mediation models

**Figure 1:**
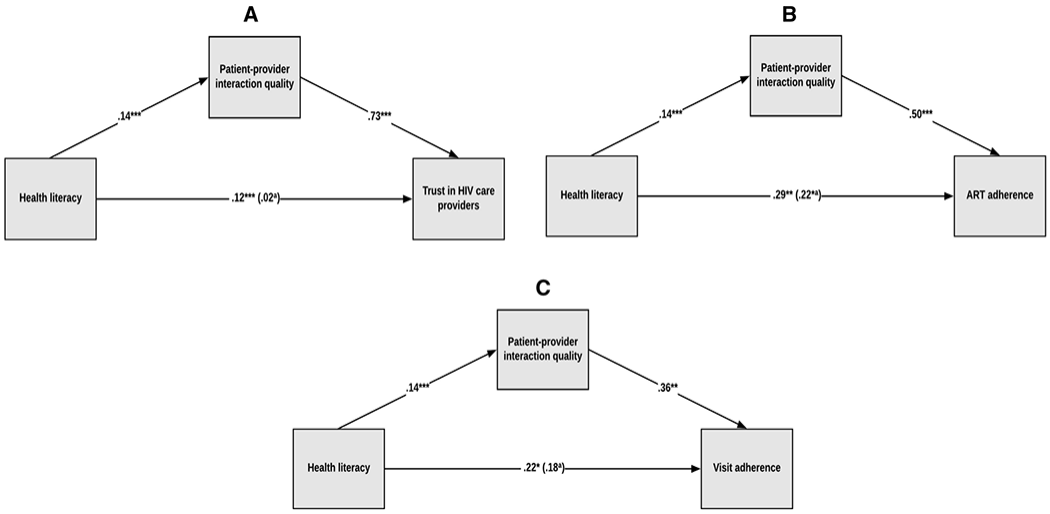
A-C: The indirect effect of health literacy on trust in HIV care providers, ART adherence, and visit adherence through patient-provider interaction quality. *p < .05, **p < .01, ***p < .001. ^a^When patient-provider interaction quality is in the model.

We tested three mediation models depicted in Figures 1A–C, where health literacy leads to patient-provider interaction quality, which in turn leads to trust in HIV providers, ART adherence or visit adherence.

The total effect of health literacy on trust in HIV care providers was significant (B=.12, SE=.02, p=.000), suggesting that higher health literacy is associated with higher trust in HIV providers. The indirect effect of health literacy on trust in HIV care providers was also significant (B=.10, SE=.01, CI[.071, .128]), suggesting that higher health literacy leads to higher perceived patient-provider interaction quality, which in turn leads to higher trust in HIV care providers. The total effect of health literacy on ART adherence was also significant (B=.29, SE=.09, p=.001), suggesting that higher health literacy is associated with higher ART adherence. The indirect effect of health literacy on ART adherence was also significant (B=.07, SE=.02, CI[.032, .114]), indicating that higher health literacy leads to higher patient-provider interaction quality, which in turn leads to higher levels of ART adherence. Similarly, the total effect of health literacy on HIV primary care visit adherence was significant (B=.22, SE=.09, p=.014), suggesting that higher health literacy is associated with greater visit adherence. The indirect effect of health literacy on visit adherence was also significant (B=.05, SE=.02, CI[.011, .093]), indicating that higher health literacy is associated with higher patient-provider interaction quality, which in turn leads to fewer missed visits.

### Integration of qualitative and quantitative findings

The quantitative mediation analyses presented in the three figures complement the qualitative thematic findings. Themes 1 and 2 focused on aspects of the quality of provider communication, specifically the positive effects of respect and non-verbal cues and the negative effects of providers’ expressions of condescension and judgement. Theme 3 centered on impacts of patient health literacy. Theme 4 focused on how provider communication influences trust. Quantitative mediation analyses indicated that higher health literacy (Theme 3) is associated with higher patient-provider interaction quality (Themes 1, 2, and 4), which in turn is associated with higher levels of trust in HIV providers (Theme 4), as well as continuum of care outcomes, namely improved antiretroviral medication adherence and reduced missed visits. While the qualitative data reflect the importance of good provider communication and their own health literacy as important aspects of care for women’s care satisfaction and well-being from their own perspectives, the quantitative results also link these factors to important HIV-related health behaviors. Qualitative and quantitative findings together suggest that improving provider communication and patient health literacy could have a positive impact on provider trust and women’s outcomes along the HIV continuum of care.

## DISCUSSION

Clear, unambiguous, and respectful provider communication, as well as improved health literacy are essential to quality healthcare.^36^ Through our qualitative analysis, we uncovered gaps in communication that may lead to serious medical consequences, particularly as experienced by people of color living with HIV. Through our quantitative analysis, we statistically substantiated the effects of health literacy on patient’s trust of their providers, their ART adherence, and their HIV visit adherence, and suggest that these relationships work through the pathway of improving patient-provider interaction quality. While our sample only included WLHIV, these findings may also have application for patients who are not living with HIV, including those with lower health literacy and patients living with other stigmatized health conditions such as substance use disorders.

Our findings are consistent with the existing literature on patient-provider communication and people living with HIV in that we found gaps in communication related to the use of stigmatizing language.^2,4,37^ In contrast, we found that when patients experienced positive communication, they expressed greater trust toward their provider. This is noteworthy, because prior studies have shown that improved trust is associated with better patient outcomes across the HIV continuum of care,^2,38,39^ which was also evident in our quantitative analysis.

One pathway to address these gaps in communication and health literacy may be to promote shared decision-making in the delivery of HIV-related care. The topic of shared decision-making is becoming more prominent in discourse related to improving provision of care for underserved populations. Shared decision-making occurs when patients and providers collaborate to determine the best course of action for the patient’s care, using clear respectful communication and sharing information that considers patient health literacy level. Evidence has shown that the practice of shared decision making in clinical settings improves communication between providers and patients.^40^ Since, our sample had low health literacy and reported examples of poor provider communication, adopting the shared decision making model in HIV care settings – where providers and patients actively engaged with one another to determine the ideal care plan – could address the effects of both provider communication and patient health literacy.

### Limitations

Qualitative data collected from focus groups and interviews can be subject to social desirability biases. Participants were recruited from a longitudinal cohort study, meaning that participants were already familiar with research and may have been more actively engaged in HIV care as compared to their peers. Since our sample was older, with an average age over 50 years, their reported experiences may be more common among older adults rather than young adults, with many having lived with HIV for a number of years. This is important, because these women may have been more comfortable with and knowledgeable about their diagnosis, and yet, they still expressed frustrations with poor communication in their healthcare settings. Our quantitative analyses were based on cross-sectional data and thus cannot provide evidence of directionality or causality.

## CONCLUSIONS

Study findings underscore the need for more effective provider communication, identification of ways to enhance and accommodate patient health literacy, and both constructs’ effects on patient’s trust in her provider and her HIV continuum of care outcomes. Guidelines, recommendations, and interventions, such as the adoption of shared decision-making in clinical settings, can be used to promote higher quality provider-patient interactions potentially leading to improved clinical outcomes.^21,26,37,41–44^
